# The path to becoming a clinical or radiation oncologist in Nigeria

**DOI:** 10.3332/ecancer.2022.1440

**Published:** 2022-08-11

**Authors:** Ifeanyichukwu Augustine Onyeodi, Gabriel T Fagbenro, Mariam A Bashir, Opeyemi M Awofeso, Adedayo O Joseph

**Affiliations:** 1 Department of Community Health & Primary Care, College of Medicine, University of Lagos, 103, Ilaje Road, Bariga, Lagos 100254, Nigeria; 2 Lagos University Teaching Hospital, Idi-Araba, Lagos 100254, Nigeria; 3 St. Nicholas Hospital, Lagos Island, Lagos 101001, Nigeria; 4 NSIA-LUTH Cancer Centre, Lagos 100254, Nigeria

**Keywords:** career, pathway, clinical oncology, radiation oncology, training

## Abstract

The number of cancer patients in Nigeria continues to rise; however, global advances in cancer research are making the provision of optimal care possible. Cancer management is globally agreed to be multidisciplinary, with patients now having the right to benefit from progress in systemic cancer therapy and radio-diagnosis by receiving treatment from adequately trained and highly skilled clinical and radiation oncologists. Radiation oncology is part of the three divisions that make up oncology – medical oncology, surgical oncology and radiation oncology. This discipline in recent times has been developed into Clinical Oncology and although both clinical oncologists and medical oncologists continue to deliver non-surgical cancer treatment, only clinical oncologists are qualified to deliver radiotherapy in the management of cancers. Though clinical oncologists continue to provide quality cancer workforce for the country’s increasing number of cancer patients, much is still unknown about this discipline in Nigeria. It is hoped that inspiring radio-oncologists will take note of the information provided by this article as a guide. This paper chronicles the multifarious process involved in training to become a clinical and radiation-oncologist in Nigeria, plus the requirements, as well as pertinent information a budding physician seeking to advance in this highly specialised field requires.

## Introduction

Cancer is reported to be the second leading cause of death globally, and single-handedly was responsible for an estimated 9.6 million deaths in 2018 [1]. With approximately 70% of these cancer deaths occurring in low- and middle-income countries such as Nigeria, one way of reducing the cancer burden besides avoiding risk factors and implementing existing evidence-based prevention strategies, is the training of adequate and competent cancer specialists who will be well-versed in various treatment options for cancer patients [2].

Management of cancer patients often involves a combination of several treatment modalities. Some specialists whose expertise are sought in cancer management include the surgical oncologist who removes the tumour, the medical oncologist who delivers systemic therapy such as chemotherapy, hormone therapy and immunotherapy and the clinical oncologist who in addition to being able to administer non-surgical cancer treatment, is also qualified to administer radiotherapy [3].

Postgraduate medical education in clinical oncology is one involving various learning activities for doctors to develop relevant clinical knowledge and skills appropriate to clinical and radiation oncology treatment after completion of basic medical education. The training is aimed at raising specialists who have sound understanding of core radiobiological principles and are able to disseminate and translate this knowledge into high-level medical practice in oncology for the benefit of patients [4, 5].

Unfortunately, there is a lack of literature about postgraduate medical education in clinical oncology in Nigeria. Not much is known about this speciality amongst many medical graduates in Nigeria due to reasons such as the limited number of training facilities, and in schools where clinical-oncology residents are trained there is little exposure to this speciality hence many graduate without realising the potential in this speciality. This gap in the literature is also challenging for medical graduates who would like to become clinical oncologists in Nigeria and contribute to the documented understaffed number of clinical oncologists in the country [6, 7].

This article for medical students and graduates who have had little to no exposure about clinical oncology serves to bring them to the realisation that clinical oncology is the new frontier with endless possibilities and also attempts to chronicle the pathway to becoming a clinical oncologist in Nigeria, identify existing training, perceived challenges to the training as well as make recommendations.

## Oncology

Oncology refers to the study of cancer. Doctors who treat cancer and provide medical care for persons diagnosed with cancers are called oncologists [8]. The field of oncology is largely divided into three major areas: the medical oncologist who treats cancer using chemotherapy or other medications, such as targeted therapy or immunotherapy. The surgical oncologist responsible for removing tumours as well as performing certain types of biopsies to help diagnose cancer and the radiation oncologist who treats cancer using radiation therapy [8]. Some countries like the UK and Nigeria have since had the radiation oncology discipline metamorphosed into clinical oncology. Hence, these groups of physicians also double as doctors who can administer various systemic therapies for cancer treatment [3, 5].

Globally, cancer has been reported as a major source of morbidity and mortality [9]. In 2008, there were 12.7 million new cases and 7.6 million cancer-related deaths [10] and this number increased by 2 million in 2018 [1]. Nigeria’s population is estimated to be about 193 million and a 3.2% annual growth rate has been predicted [10]. Studies have reported about 100,000 new cancer cases occurring yearly with high case fatality [11] and like most developing countries, we are plagued often with late presentations often leading to poor prognosis, inadequate number of specialists and limited number of comprehensive cancer care facilities [7].

With the majority of the newly reported cancer cases occurring in developing countries and with a projected 70% of all new cases of cancer occurring in developing countries by 2030 [12], it is important that our cancer workforce be critically analysed and suggestions rendered to make it better prepared for the challenges ahead. This continued prevalence of cancer means that there is an ongoing need for a committed workforce in cancer within the medical profession. With the economic and social development status of a country correlating closely with the burden of cancer and the shortage of human resources, it has become pertinent to address the shortage of clinical oncologists as well as other workforces in cancer care if we must meet to the sustainable development goals for non-communicable diseases by 2030 [13].

### Clinical and radio-oncologist

A clinical oncology specialist is one involved in any type of cancer treatment that is not surgery, including radiotherapy and systemic therapies [3]. This highly specialised group of experts treat patients with various forms of radiotherapy, systemic chemotherapy, biological agents and hormone therapy. Radiotherapy treatment by a clinical oncologist often involves using radiation energy from machine-generated particles and radioactive materials for precisely targeting and killing tumours. This is possible with the help of machines such as cobalt 60 and linear accelerators (LINACs) which allow X-ray beams and proton beams into the body [3]. Clinical oncologists also offer brachytherapy which involves using radioisotopes to destroy tumour cells as seen with the use of radioiodine to treat thyroid cancer and iridium to treat cervical malignancies [14]. Systemic therapies include chemotherapy, hormone therapy and immune-therapy. They also are included in the longlist of treatment clinical oncologists offer. Systemic therapies are indicated in different conditions [15]. While chemotherapy is used to stop cancer cells from multiplying, hormone therapies are used in tumours which respond to hormones and immunotherapy is employed to prime the body’s immune system to fight cancer. These therapies can be applied either alone or in combination [3].

Inarguably, access to affordable cancer treatment offered by clinical oncologists is a serious issue and has been reported to be a major impediment to global cancer control [16]. However, the enormous challenge of inadequate availability of health care professionals also confronts this discipline and contributes to cancer health disparities.

The profound shortage of oncologists in several parts of the world has been documented. A shortage of less than 2,300 medical oncologists is expected by 2025 in the United States [17]. A global survey on clinical oncology workforce revealed that countries in Europe, North-America and South-America had a better clinical-oncology workforce than many African countries. Egypt was notably the only exception in Africa with 1,500 clinical oncologists [18–20]. Sadly, some countries in Africa and Asia such as Burundi, Central African Republic and Afghanistan have faced an extreme shortage of clinical oncologists as some had less than 1,000 incident cancers per clinical oncologist, there was no clinical oncologist in many others. Many countries in Europe, North America and South America fared better with clinical oncologists providing care for <150 patients with a new diagnosis of cancer in these countries [21] Although there is no national database of clinical oncologists in Nigeria, some articles report a total number of 70 clinical oncologists [22]. Although the magnitude of this problem is poorly described in the available literature, the size of the problem however can be estimated from the $400,000,000 lost by Nigeria to medical tourism on cancer [23].

### Why consider clinical oncology as a speciality?

#### Collaborative speciality

For medical graduates’ intent on collaboration, clinical oncology may just be the right area of specialisation for them as cancer management today has evolved from the days when physicians managed cancer patients autonomously. Cancer management today requires multidisciplinary teams involving different professionals ranging from doctors, nurses, pathologists, medical oncologists, radio-oncologists and social workers all working together to create a patient’s overall treatment plan [8]. This speciality affords one an opportunity to work closely and learn to respect the expertise of professionals outside medicine such as physicists, programme developers, technology experts, pharmaceutical companies – most of whom you wouldn’t readily work with if you were in other training.

#### Opportunity to work with cancer patients

Cancer is now a common disease. Countries like the UK estimate that about 1 in 2 persons in the UK will likely be diagnosed with a malignancy in their life-time [24] and here in Nigeria, there are estimated 100,000 cases occurring yearly [11]. This means that many people will have the experience of a close friend or family member being diagnosed with cancer. Clinical oncology serves as that speciality that enables medical graduates who may or may not have personal stories with cancers but would like the opportunity to work closely with cancer patients. It affords the opportunity to be able to change the course of the disease for patients with cancer, and when cure is not possible, provide effective palliation as well as improve the quality of life remaining [25]. This experience has been reported by many involved in oncology to be very rewarding [26].

#### Constantly improving technology and treatments

For medical graduates interested in technology and the science behind radiobiology and physics, clinical oncology is the field for you as not many medical specialities allow to such a large extent the option of incorporating cutting-edge technology into your practice as clinical oncology [27]. Clinical oncologists are involved in radiotherapy planning sessions which require practical, three-dimensional thinking to target cancer cells while sparing normal tissue.

#### Research potential

The clinical oncology speciality is one that is research driven. Novel clinical trial opportunities are available for the treatments of different cancers to encourage the development of effective treatments [3, 28]. This rapidly evolving research in oncology ensures oncologists continually update their knowledge and skills in cancer advancements to be able to offer the best services to their patients [29].

#### Financially rewarding

Although a medical graduate is advised against hinging their future medical speciality training on financial renumeration, it can be assuring for intending clinical oncologists that this speciality is financially rewarding. In a 2019 Medscape physician compensation report, oncologists were reported to be above the middle earners of all physician specialities [30]. Plus, like other medical doctors, oncologists can also earn extra income by teaching, speaking at engagements and publishing articles or books [31].

#### Development of good communication skills

This speciality affords you the opportunity to develop good communication skills which are essential to patient management and teamwork. Working practice in oncology requires regular liaising with many different specialities, so provides the clinical oncologist with an opportunity to develop good communication and inter-personal skills [32].

#### Cons of clinical oncology

One daunting challenge that has been recognised among clinical oncologists as also reported among others in the field of oncology is the emotional challenge that comes with the job. With cancer being the second leading cause of death [2], this means that sometimes patients will be lost and this can be very difficult to handle. In oncology, there is also the struggle of being helpless as in some cases oncologists are unable to do anything for some cancer patients, following the failure of science and despite giving it your best [25, 26].

#### Clinical and radiation-oncology in Nigeria

Historically, clinical oncologists began as radiotherapists [33]. Since January 1896, when the world first recorded the first therapeutic use of ionising radiation following its usage in treating breast cancer in Chicago, radiotherapy has undergone tremendous advancements and has become a treatment modality for a lot of malignancies and diseases [34].

Radiotherapy practice in Nigeria dates back to 1968 when Nigeria’s first orthovoltage machine was acquired at the Lagos University Teaching Hospital (LUTH) – Nigeria’s first radiology department [35]. This was the first superficial radiotherapy machine in Nigeria and by 1973, LUTH installed a telecobalt – Nigeria’s first megavoltage radiotherapy machine [35].

The earliest radiotherapy centres in Nigeria include LUTH, Ahmadu Bello University Teaching Hospital (ABUTH), Zaria and University College Hospital (UCH), Ibadan, and, by 2001, the number of radiotherapy centres in Nigeria increased to five – LUTH, UCH, ABUTH, National Hospital Abuja (NHA) and Eko Hospital with all of them having a total of only six megavoltage teletherapy machines [36]. This number of radiotherapy centres further increased to nine by the end of 2010 with all of them having a total of eight megavoltage machines [21]. There has also been documented increase in the number of radiotherapy centres and equipment in countries in Africa, and regions outside Africa [37, 38]. This rise in the number of radiotherapy facilities across the world could be attributed to the increasing global cancer incidence and the new found acceptance of radiotherapy as an integral part of cancer treatment [33, 39].

Research findings on Nigeria’s available technology for radiotherapy reveal that by 2010, only three telecobalt machines were operational in Nigeria in LUTH, ABUTH and Eko Hospital [35]. However, at the end of 2015, only two of this telecobalt machines which were situated in ABUTH and Eko hospital were functionally operational [21]. Nigeria’s first LINAC was installed about 10 years ago at the NHA in the Federal Capital, Abuja. Since then, the federal government has also acquired LINAC’s for LUTH, ABUTH, the University of Benin Teaching Hospital, University of Nigeria Teaching Hospital, Enugu (UNTH) and Usman Danfodiyo University Teaching Hospital [21].

With regard to brachytherapy, by the end of 2013, there were only six brachytherapy units in Nigeria which were located in five of the nine radiotherapy centres. Four of which were low-dose rate (LDR), while two are high-dose rate (HDR) brachytherapy [35]. This number reduced to four by 2019 – two LDR, two HDR with only three being functional [21].

Nigeria’s attempt at its first advanced radiotherapy and chemotherapy was birthed on the 1 May 2019 following a structured public-private partnership (PPP) arrangement between the Nigeria Sovereign Investment Authority (NSIA) and the LUTH [40]. The edifice covers over 1,500 m^2^ and has three LINACs already installed, one halcyon, and two vital beams. It boasts of a well-equipped robust treatment planning system, a brachytherapy machine to treat cervical and prostate cancer and a chemotherapy suite that can accommodate as many as 15 patients at any time [41]. The project was an estimated US$11 million investment and plans are underway to replicate this project across the country.

No literature could be found on the first generation of Nigerian clinical oncologists but what is known about clinical oncology like other medical specialists is that Nigerian medical specialists were largely trained abroad, mainly in England, Scotland, Ireland and the United States of America until the postgraduate medical colleges were established in Nigeria. The establishment of these local training programmes over time has reduced our reliance on expatriates [33, 42] and has also helped stem the emigration of physicians [43, 44]. Some obtained additional fellowships subsequently.

Internet search revealed some notable clinical oncologists in the country. The first Nigerian to qualify as a clinical oncologist is unknown; however, Professor F.A Durosinmi-Etti [Order of the Federal Republic (OFR)] is one of the most decorated clinical oncologists in the country.

## Clinical and radiation-oncology training in Nigeria

When compared to her population size, Nigeria has limited oncology training facilities and radiotherapy treatment centres. Research has shown that as at 2009, Nigeria could only boast of eight radiation oncology centres, seven of which are owned by government and one by a private centre. Unfortunately, 90% of these centres are not fully functional [21]. Except a few of them like the NHA and the NSIA-LUTH Cancer Center [45], most of the available centres are found operating without modern-age technology, treatment planning systems and continue to suffer challenges of manpower shortages and infrastructure breakdown [20].

Nigeria suffers a lack of health care specialists and oncology is not left out. This limited number of specialists in cancer management in Nigeria is quite alarming taking cognisance of cancer incidence, prevalence and population size. Nigeria has less than 70 consultant radiation and clinical oncologists. Hence, the continuous need for further exposure of medical graduates to this speciality so that the clinical workforce in oncology in Nigeria can be increased in the future.

The journey to becoming a radiation oncologist in Nigeria begins with attending an accredited medical school and becoming licensed to practice Medicine in Nigeria. This undergraduate medical education will last a minimum of 6 years for students who are admitted via the Unified Tertiary Matriculation Examination route or a minimum of 5 years for those with a medical related first degree admitted via the direct entry route. This medical school training will culminate in the award of an MBBS or MBChB or MBBCh degree on completion [47]. After becoming a qualified medical practitioner, one is also required to be fully registered with the Medical and Dental Council of Nigeria. This will be followed by the satisfactory completion of the National Youth Service Corps (NYSC) programme or due exemption from it by the NYSC directorate.

Primary examinations into radiation and clinical oncology in Nigeria are conducted by recognised postgraduate colleges. To be certified a radio-oncologist in Nigeria, intending physicians must sit for examinations of either or both the National Postgraduate Medical College of Nigeria and the West African College of Surgeons and pass these entrance examinations [48]. Historically, clinical oncologists began as radiotherapists and because they share common ground with radiologists, they are found in the same faculty in most countries until recently where the need for specialisation has necessitated a creation of a distinct faculty [33] However, in some postgraduate medical colleges (West African College of Surgeons (WACS), National Postgraduate Medical College of Nigeria) they are still found within the faculty of radiology.

A full-time Residency Training Programme in radiation-oncology is offered in a few hospitals in Nigeria. These programmes lead to a fellowship of the National Postgraduate Medical College of Nigeria or the Fellowship of West African College of Surgeons/Physicians. Some accredited training centres include [21, 35]:

- University College Hospital, Ibadan (www.com.ui.edu.ng/index.php/radiation-oncology)
- Lagos University Teaching Hospital, Idi-Araba (www.luth.gov.ng)
- Lagos State University Teaching Hospital, Ikeja (www.lasuth.org.ng)
- National Hospital Abuja (www.nationalhospital.gov.ng/department-services/department-of-radiotherapy-and-oncology)
- The University of Nigeria Teaching Hospital, Nsukka (www.unth.org/clinical-departments/radiotherapy-clinical-oncology)
- Ahmadu Bello University Teaching Hospital, Zaria (www.abuth.org.ng/27-departments/clinicals-depts/34-oncology)
- Usman Danfodiyo University Teaching Hospital (www.uduth.org.ng)
- The University of Benin Teaching Hospital (https://ubth.org/clinical-departments/radiotherapy-clinical-oncology)
- Federal Medical Centre Gombe, Gombe State, Nigeria

The year of the first set of NPMCN and WACS clinical oncology residents could not be found in the available literature. However, it is now known that both the West-African and national postgraduate medical colleges conduct primary examinations for radiation oncology residents biannually. The primary entrance examinations comprise multiple-choice questions. The exam often consists of multiple-choice questions in the basic medical sciences – anatomy, physiology and pathology and O-level general physics (mechanics, light, optics, electricity, magnetism, atomic physics, etc.). After passing the primary examination, prospective candidates will have to submit to a hospital-based interview which may be written or oral. These interviews are conducted independently by each accredited clinical-oncology training centre, after which continuous physical presence of at least 12 months in the facility. The number of intakes varies among the different training facilities. The residents will undergo their training in these facilities while submitting to either of the postgraduate college exams.

Eligibility for the part 1 membership examination under the WACS postgraduate medical college requires a continuous physical presence of at least 12 months in an accredited radiotherapy department as a clinician. Candidates are often tested on their Basic Sciences knowledge: Applied Physics, Radiobiology, Anatomy and Cancer Biology [5]. After candidates have passed the part 1 examination; they will be required to complete at least 36 months (including the first year) of instruction and training in full-time posts in a recognised Department of Radiotherapy and Oncology, among other requirements, before they are eligible to attempt the part 2 fellowship examination and this exam will examine their knowledge on surgical principles, medical and radiation oncology and applied pathology. Fellowship Examination in clinical oncology dwells on the candidates’ research development and ability to practice oncology unsupervised, and eligibility to sit for this exam requires a minimum of 5 years of training and a passing grade in the part 2 examination [5]. Eligibility for the part 1 membership examination under the National Postgraduate Medical College requires writing the part 1 examination after 2 years of training while the final fellowship exams are written after 2 to 3 years.

## A working-week of a Nigerian radio-oncologist

The typical working week is varied and is spent in outpatient clinics – where new patients are seen, and old-patients are further reviewed, ward rounds, chemotherapy planning sessions, brachytherapy, planning radiotherapy and attending multidisciplinary team meetings [3, 49].

Although clinical trials in oncology remain low in Nigeria due to poor infrastructure, inadequate funding and lack of experienced personnel to execute clinical trials, clinical oncologists in Nigeria like their counterparts worldwide also contribute to research through clinical trials or translational research [24, 50]. A notable example is the ARETTA (Assessing Response to Neoadjuvant Taxotere and Trastuzumab in Nigerian Women With HER2-positive Breast Cancer) study, an ongoing clinical trial assessing the response to trastuzumab and taxanes among black women with early-stage breast cancer patients.

## Challenges to radio-oncology residency training in Nigeria

A peculiar problem for radio-oncology residency training in Nigeria is the constantly evolving technological advancement in field witnesses hence the constant need for training facilities to be up to date [6, 51]. Training facilities still suffer from a lack of treatment planning systems, modern LINAC, CT simulator and Mosaiq for R&V [33].

Many training centres also suffer from a lack of high-tech machines such as CT scanners and MRI Scanners. Some training centres in Nigeria can only boast CT scanners with four slices while only a few centres have 64-slice or 128-slice scanners. This problem is also seen with MRI scanners in Nigeria as some centres still use MRI Scanners of low-field strength (0.2–0.3 Tesla) units [37, 52]. The rapidly evolving medical education and technology in clinical oncology also requires that clinical oncology trainers, in addition to professional qualifications and competencies, need to be constantly trained and retrained [53].

Since its inception in Nigeria, medical physics training as far back as 1968 has continued to suffer several setbacks. Training in medical physics in Nigeria is still a far cry from what is obtainable in Europe. It was only in 2012 that the clinical training of medical physicists geared towards radiation oncology started. These medical physics education improvements have to be sustained as it remains an integral part of further boosting clinical oncology training in Nigeria [54].

## Recommendation

The foregoing implies that clinical oncology residency training in Nigeria needs to be strengthened on many fronts. Some recommendations will be:

1. The importance of subspecialisation in clinical oncology – Many articles have argued the commencement of subspeciality training in clinical oncology [50, 55, 56] . Specialisation in clinical oncology is necessary due to the complexity of diagnostic and therapeutic radiology, and it also serves to increase proficiency among clinical oncologists.
2. Need for overseas clinical training – Clinical oncology training in Nigeria is still in its formative years and would require a greater international outlook and acceptability. This can be achieved by inculcating overseas clinical observership and training and getting foreign examiners from respected postgraduate medical colleges to partake in our fellowship exams.
3. More PPPs – Replication of PPP agreements such the NSIA-LUTH Cancer Center to encourage the creation of top-notch cancer centres where residents can be adequately trained. These partnerships have proved necessary in running the cancer centres sustainably [57].

## Conclusion

There is no doubt that clinical oncology speciality training in Nigeria is still within its formative years. Going forward, a lot needs to be done to reposition it. Clinical oncology, however, remains the new frontier of medicine. For medical graduates interested in pursuing an exciting career with new therapeutic opportunities, great research potential and the chance to impact cancer patients’ lives significantly, clinical oncology remains the best bet!

## Source of funding

No funding was received for this work.

## Conflicts of interest

The authors have declared that they have no competing or potential conflicts of interests,

## Authors’ contributions

**Table:** 

Dr Onyeodi Ifeanyichukwu A.	Conception, design, literature review, manuscript writing, manuscript revision
Dr Fagbenro Gabriel T.	Conception, design, manuscript writing, manuscript revision
Dr Bashiru Mariam A.	Conception, design, literature review, manuscript writing, manuscript revision
Dr Awofeso Opeyemi M.	Conception, design, manuscript writing, manuscript revision
Dr Adedayo Joseph O.	Conception, design, manuscript writing, manuscript revision
All the authors have read and agreed to the final manuscript.	
